# The A/T/N biomarker scheme and patterns of brain atrophy assessed in mild cognitive impairment

**DOI:** 10.1038/s41598-018-26151-8

**Published:** 2018-05-30

**Authors:** Urban Ekman, Daniel Ferreira, Eric Westman

**Affiliations:** 1 Division of Clinical Geriatrics, Department of Neurobiology, Care Sciences, and Society, Karolinska Institutet, Stockholm, Sweden; 2 Department of Neuroimaging, Centre for Neuroimaging Sciences, Institute of Psychiatry, Psychology and Neuroscience: King’s College London, London, UK

## Abstract

The objective of this study was to evaluate the A/T/N biomarker scheme in relation with brain atrophy patterns in individuals with mild cognitive impairment (MCI). Of the 154 participants with MCI, 74 progressed to AD within 36-months, and 80 remained stable. In addition, 101 cognitively healthy participants and 102 participants with AD were included. The A/T/N classification was assessed with cerebrospinal fluid markers. Each individual was rated as either positive (abnormal) or negative (normal) on each biomarker. Brain atrophy was assessed with visual ratings from magnetic resonance imaging. None of the individuals with MCI progressed to AD if they had a negative “A” biomarker in conjunction with minimal atrophy. In contrary, several individuals with MCI progressed to AD if they had a positive “A” biomarker in conjunction with minimal atrophy. Numerous individuals with MCI showed inconsistency in the neurodegeneration domain (“N”) regarding t-tau and atrophy. The assessment of the A/T/N classification scheme in addition with brain atrophy patterns in MCI, increases the knowledge of the clinical trajectories and the variability within the neurodegeneration domain. This emphasises that individuals with MCI display heterogeneous longitudinal patterns closely connected to their biomarker profiles, which could have important clinical implications.

## Introduction

The clinical manifestations of mild cognitive impairment (MCI) are associated with diverse trajectories^1–3^. The addition of biomarkers might enhance predictions of the longitudinal development from MCI to Alzheimer’s disease (AD)^4,5^. The A/T/N binary biomarker classification scheme has recently been proposed, aiming to be easily applicable on an individual level^6^. In keeping with the National Institute on Aging Alzheimer’s Association (NIA-AA)^7^ and the International Working Group (IWG)^8^, the “A” class corresponds with an amyloid beta (Aβ) biomarker; the “T” class with a tau biomarker; and “N” with a neurodegeneration biomarker. Cerebrospinal fluid (CSF) biomarkers have the advantage that all A/T/N categories can be measured. Another way of assessing the “N” domain is to visually rate atrophy in the brain using rating scales for magnetic resonance imaging (MRI). Visual rating scales are commonly applied in specialized clinical settings^9^ and allow identification of different AD-related patterns of atrophy^10–13^. The patterns of atrophy resemble the distribution of neurofibrillary tangles (NFT)^14^ in the brain. This may help to reach a greater understanding of the mechanisms underlying heterogeneity and disease progression in AD. Translating atrophy patterns related to AD-subtypes and the A/T/N classification scheme to patients with MCI is of utmost importance. We evaluated the A/T/N classification scheme using CSF markers in individuals with MCI that either progressed to AD or remained stable. In addition, we studied brain atrophy patterns generated from visual rating scales in combination with the A/T/N classification, with a special focus on variability in the “N” domain.

## Methods

### Study population

Participants were included from the Alzheimer’s Disease Neuroimaging Initiative (ADNI), which was launched in 2003 by the National Institute on Aging, the National Institute of Biomedical Imaging and Bioengineering, the Food and Drug Administration, private pharmaceutical companies, and non-profit organisations^11^. Only data from the ADNI-1 cohort were included in the current study. The clinical diagnostic procedure has previously been described^15^. In summary, healthy controls (HC) had no memory complaints, and had a Clinical Dementia Rating (CDR)^16^ score of 0, normal performance on objective cognitive measures (Wechsler Memory Scale-Revised (Logical Memory II subscale; maximum score of 25))^17^, and activities of daily living (ADL). On the Mini Mental State Examination (MMSE; total score = 30) the range for HC was 24–30, and the education-adjusted cut-off score for Logical Memory II was based on education established as ≥9 for 16 years of education, ≥5 for 8–15 years, and ≥3 for 0–7 years. The subjects with MCI had memory complaints, a CDR score of 0.5, mild impairments on objective cognitive measures (i.e., amnestic deficits), and no significant impairments in ADL. On MMSE the range for MCI was 24–30, and the education-adjusted cut-off score for Logical Memory II was ≤8 for 16 years of education, ≤4 for 8–15 years, and ≤2 for 0–7 years. The participants with MCI did not qualify for the diagnosis of AD. Finally, subjects with AD had mild AD with memory complaints, a CDR score ≥0.5, and  significant impairments on objective cognitive measures and in ADL. On MMSE the range for AD was 20–26, and the education-adjusted cut-off score for Logical Memory II was ≤8 for 16 years, ≤4 for 8–15 years, and ≤2 for 0–7 years. Individuals with AD met the National Institute of Neurological and Communicative Disorders and Stroke-Alzheimer’s Disease and Related Disorders Association criteria for probable AD^18^. Importantly, all diagnoses were independent of biomarker information. All included participants had available information on CSF biomarkers and MRI atrophy at baseline, as well as longitudinal information regarding diagnostic status. A total of 154 participants with MCI were included in the current study. Of those, 74 progressed to AD (MCI-P) within 36-months, and 80 remained stable in their diagnosis across time (MCI-S). In addition, 101 HC participants and 102 participants with AD were included for descriptive comparisons. We classified the individuals and compared the groups by assessing the baseline data. For the cognitive characterization, we conducted group comparisons of global cognitive performance as measured with MMSE, and episodic memory as measured with the delayed recall in the Auditory Verbal Learning Test (AVLT; total score = 15). Demographic and clinical variables are presented in Table 1. The study protocols were approved by the institutional review boards of all included ADNI centres (see supplementary information), and written informed consent (including extensive description of the ADNI) was acquired from all included participants according to the Declaration of Helsinki. All study methods and protocols were performed in accordance with the relevant guidelines and regulations.

**Table 1:** Demographics and clinical variables.

	HC (n = 101)	MCI-S (n = 80)	MCI-P (n = 74)	AD (n = 102)
Age	75,46 (5,18)	74,49 (7,15)	74,51 (7,29)	74,96 (7,91)
Years of education	15,61 (2,90)	16,15 (2,89)	15,66 (3,13)	15,13 (3,29)
MMSE*	29,05 (1,05)	27,19 (1,66)	26,49 (1,82)	23,55 (1,89)
AVLT delayed*	7,19 (3,55)	3,33 (3,29)	1,42 (2,04)	0,95 (1,94)
Aβ_42_*	206,86 (53,78)	177,92 (57,94)	147,44 (37,97)	142,46 (39,61)
p-tau*	24,83 (13,96)	30,58 (15,72)	40,35 (16,70)	41,76 (19,77)
t-tau*	69,12 (27,89)	85,96 (43,21)	115,77 (56,84)	121,52 (57,45)
APOE ε4 positive N/Y*	76/25	46/34	27/47	31/71
-Sex W/M	49/52	26/54	28/46	43/59

Means, parentheses = standard deviations. HC = healthy controls. MCI-S = MCI participants that are clinically stable across 36 months of follow-up. MCI-P = MCI participants that progress to AD within 36 months of follow-up. AD = Alzheimer’s disease. MCI = mild cognitive impairment. MMSE = Mini Mental State Examination. AVLT = Auditory Verbal Learning Test. p-tau = phosphorylated tau. t-tau = total tau. N = No/Y = Yes. W = Women. M = Men. *p < 0.01.

### Assessment procedure for cerebrospinal fluid biomarkers

For CSF, the Aβ_42,_ phosphorylated tau (p-tau), and total tau (t-tau) protein levels were measured from the ADNI baseline aliquots (See Shaw *et al*., 2009 for detailed information)^19^. In summary, calibration curves were produced for each CSF marker using aqueous buffered solutions that contained the combination of the three CSF markers at concentrations ranging from 56 to 1,948 pg/ml for recombinant tau, 27 to 1,574 pg/ml for synthetic Aβ_42_ peptide, and 8 to 230 pg/ml for a tau synthetic peptide phosphorylated at the threonine 181 position.

### Assessment of the A/T/N classification system

The A/T/N is a three-tailed binary +/− categorization system based on the underlying pathophysiology in each category^6^. We classified the Aβ biomarker “A” with CSF Aβ_42_, the tau pathology biomarker “T” with CSF p-tau, and finally the biomarker of neurodegeneration “N” with CSF t-tau. Each individual was rated as either positive (+; i.e., abnormal) or negative (−; i.e., normal) on each biomarker.

The cut-offs of the selected CSF biomarkers have previously been published^19^. In summary, the cut-off values were derived from the comparison between ADNI AD cases and healthy controls. The individual CSF values were considered pathological (+) if ≤192 pg/ml for Aβ_42_, ≥93 pg/ml for t-tau, and ≥23 pg/ml for p-tau.

### Structural MRI and visual rating scales

For MRI, a T1-weighted magnetization-prepared rapid gradient-echo (MPRAGE) sequence was acquired on 1.5 T scanners with a voxel size of 1.1 × 1.1 × 1.2 mm^3^. All T1-weighted images were visually rated for regional brain atrophy by an experienced radiologist (See Ferreira *et al*., 2015 for detailed information)^20^. In summary, the medial temporal atrophy (MTA) scale scored the degree of atrophy from zero to four^21^. The posterior atrophy (PA) scale scored from zero to three and evaluated atrophy in the posterior cortex (posterior cingulate sulcus, precuneus, parieto-occipital sulcus, and parietal cortex)^22^. Finally, the global cortical atrophy scale – frontal subscale (GCA-F) scored from zero to three and evaluated atrophy in the frontal cortex (bordered by the central sulcus, the frontal bone, and the fissure of Sylvius)^23^. Higher scores in the three scales denote end-stage degree of atrophy. Intra-rater reliability (weighted kappa) for MTA was 0.94 and 0.89 in the left and right hemispheres respectively. Equivalent numbers for PA was 0.88, and for GCA-F 0.83.

### Classification of brain atrophy patterns

Cut-offs for the visual rating scales have previously been published^20^. In summary, normal versus abnormal cut-off points were determined for each individual in the three visual rating scales. A MTA score ≥1,5 were considered to be abnormal in the age-group 65–74, ≥2 for the age-group 75–84, and ≥2,5 for the age-group 85–94. For PA and GCA-F, a score ≥ 1 was always considered abnormal irrespective of age (age-correction did not improve diagnostic accuracy in cut-offs derivation).

**Figure 1:**
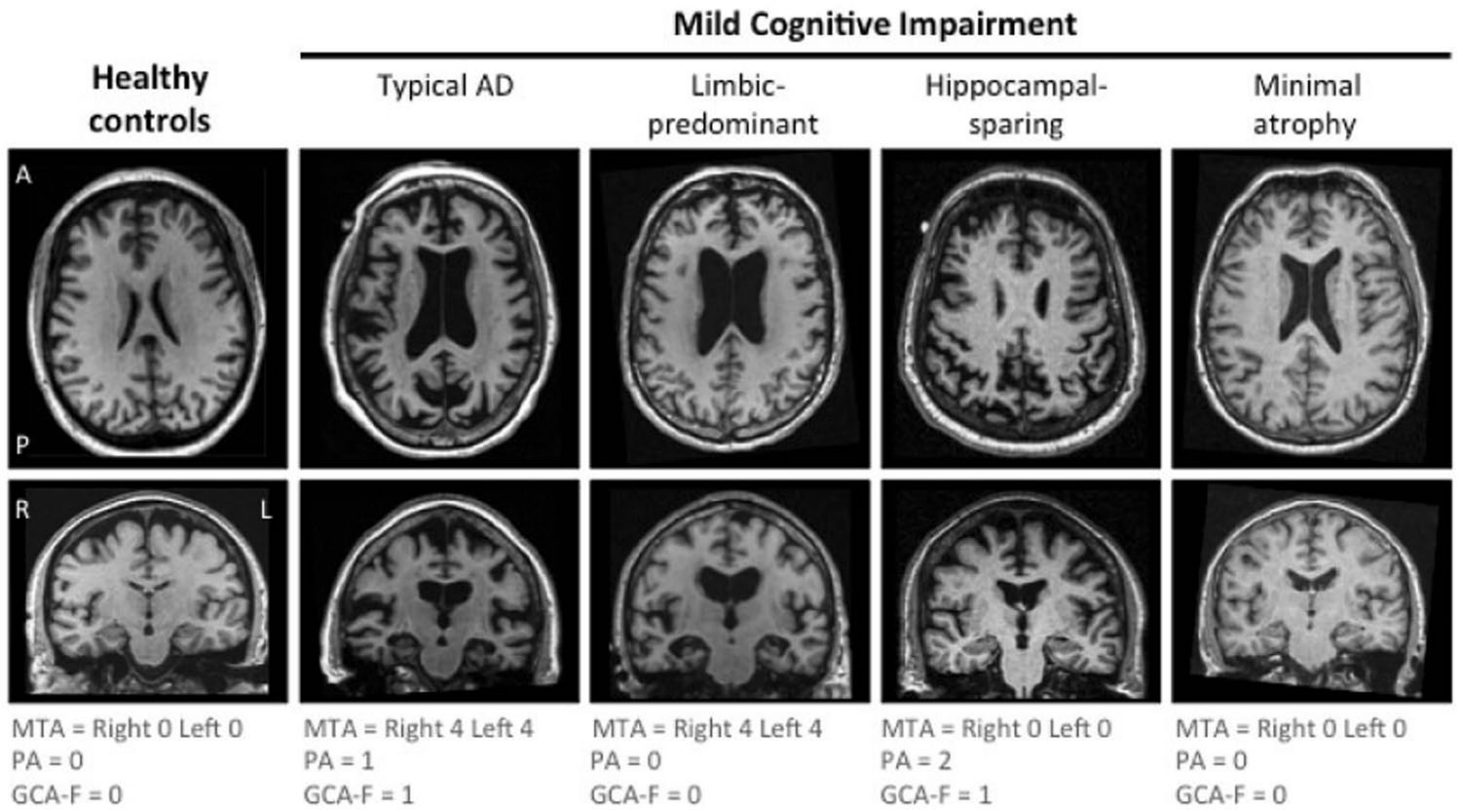
Subtypes of brain atrophy patterns in MCI from visual rating scales Atrophy was measured with the medial temporal atrophy (MTA) scale, the global cortical atrophy-frontal (GCA-F) sub-scale, and the posterior atrophy (PA) scale. The visual rating scales were based on MRI. A score of zero indicates no atrophy. A score from one to four (MTA), and from one to three (GCA-F, and PA) indicates an increasing degree of atrophy. The minimal atrophy group was defined as normal scores on all visual rating scales. The limbic-predominant group was defined as abnormal MTA and normal GCA-F and PA. The typical AD group was defined as abnormal MTA in conjunction with either an abnormal PA or GCA-F, or an abnormal MTA in conjunction with both an abnormal PA and GCA-F. The hippocampal-sparing group was defined as abnormal GCA-F and/or abnormal PA, but normal MTA. AD = Alzheimer’s disease. L = left. R = right. A = anterior. P = posterior.

The four atrophy patterns were created using the combination of MTA, PA, and GCA-F^14,24^, and have previously  been described in relation to AD^13^. The atrophy patterns for each study group are illustrated in Fig. 1. In summary, the minimal-atrophy pattern was defined as no evidence of visual brain atrophy according to the above-mentioned rating scales cut-offs. The limbic-predominant pattern was defined as an abnormal MTA, and a normal PA and GCA-F. The typical AD pattern was defined as an abnormal MTA in conjunction with either an abnormal PA or GCA-F, or an abnormal MTA in conjunction with both an abnormal PA and GCA-F. Finally, the hippocampal-sparing pattern was defined as a normal MTA and either an abnormal PA or GCA-F, or both an abnormal PA and GCA-F.

### Statistical analyses

For univariate comparisons of quantitative variables, independent one-way ANOVA/ANCOVAs were conducted with additional post-hoc follow-ups. The post-hoc analyses were Hochberg corrected for number of comparisons. Chi-square tests were conducted for categorical variables. A p-value <0.05 was deemed statistically significant. Descriptive data are presented as mean (standard deviation), and percentages. Prevalence and incidence rate for AD with a 95% confidence interval (CI) were calculated. Cox regression analyses were conducted to examine the association between A/T/N and brain atrophy characteristics in MCI at baseline and the development of AD. The Hazard ratios (HR) are presented with 95% CI and p-values. Statistical analyses were performed with the use of IBM SPSS statistics 23.

## Results

The baseline characteristics for HC, MCI-S, MCI-P, and AD are displayed in Table 1. There were no significant group differences regarding age (F_3,353_ = 0.39, p = 0.76) or years of education (F_3,353_ = 1.68, p = 0.17). The neuropsychological measures revealed group differences for global cognitive performances measured with MMSE (F_3,353_ = 19.62, p < 0.01) and episodic memory measured with AVLT delayed recall (F_3,353_ = 99.01, p < 0.01). Post-hoc analyses showed that all groups differed significantly between each other regarding MMSE and AVLT, except for MCI-P and AD that had similar performances in AVLT (p = 0.86). There were also group differences regarding the CSF biomarkers Aβ_42_ (F_3,353_ = 36.99, p < 0.01), p-tau (F_3,353_ = 22.09, p < 0.01), and t-tau (F_3,353_ = 26.01, p < 0.01). Post-hoc analyses showed that Aβ_42_ levels significantly differed between groups except for MCI-P and AD that had similar values (p = 0.98). For p-tau, post-hoc analyses showed significant group differences except for HC and MCI-S that had similar values (p = 0.13), as well as MCI-P and AD (p = 0.99). For t-tau, post-hoc analyses showed significant group differences except for HC and MCI-S that had similar values (p = 0.10), as well as MCI-P and AD (p = 0.96). The sex distribution was rather similar among groups (X^3^ = 5.11, p = 0.16). Individuals with the APOE ε4 genotype were more common in MCI-P and AD than in MCI-S and HC (X^3^ = 48.62, p < 0.01). The longitudinal examinations showed that 74 of the 154 participants (48%) with MCI progressed to AD (MCI-P) whereas 80 participants remained stable across time (MCI-S). This percentage corresponds to an incidence rate of 160 per 1000 individuals with MCI that progress yearly to AD (95% CI, 127–194).

### A/T/N assessment

**Figure 2:**
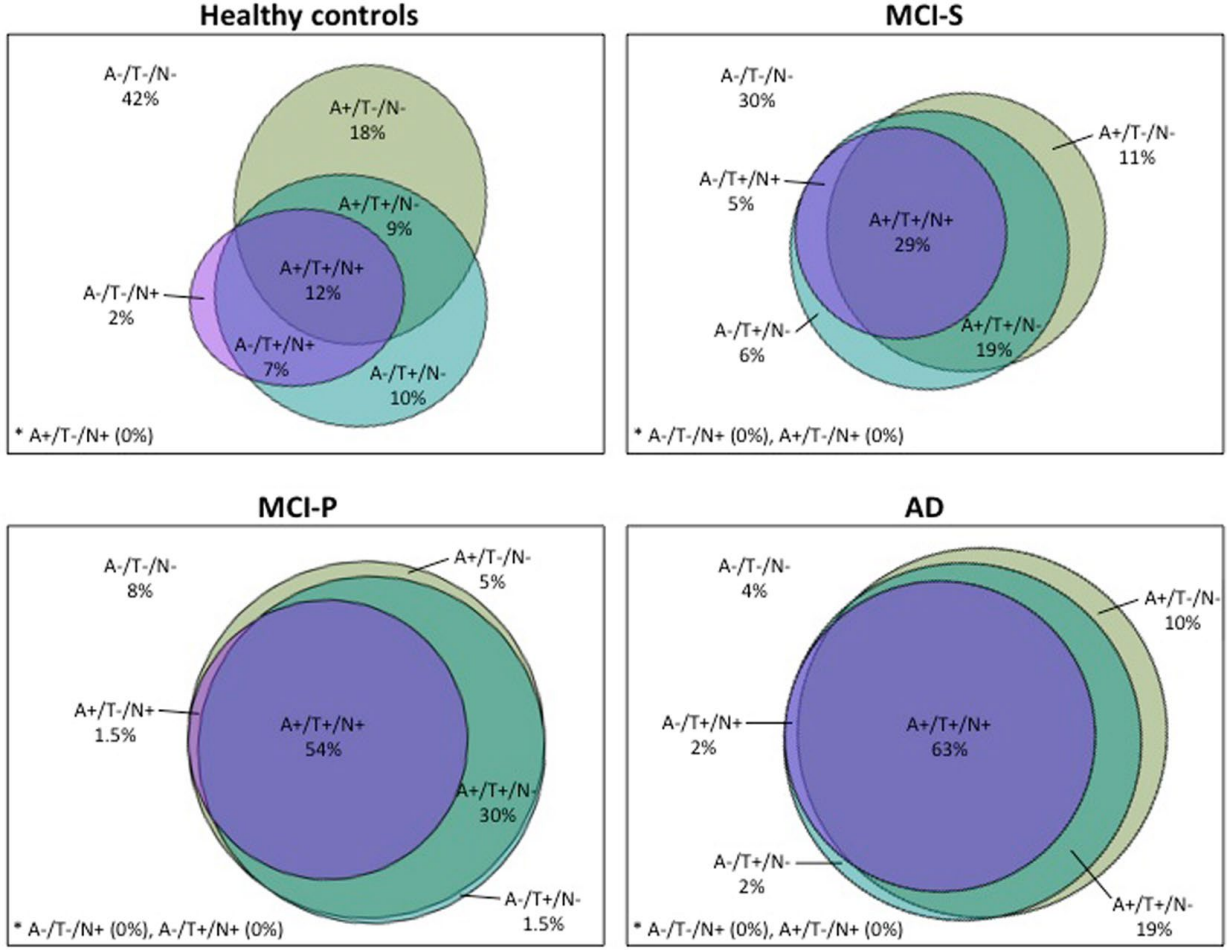
Prevalence of each A/T/N group Percentages of participants in each ATN group for healthy controls, MCI-S, MCI-P, and AD. HC = healthy controls. MCI-S = MCI participants that are clinically stable across time. MCI-P = MCI participants that progress to AD. AD = Alzheimer’s disease. MCI = mild cognitive impairment. CSF = cerebrospinal fluid. A − = CSF Aβ normal. A + = CSF Aβ abnormal. T − = CSF p-tau normal. T + = CSF p-tau abnormal. N− = CSF t-tau normal. N + = t-tau abnormal.

**Figure 3:**
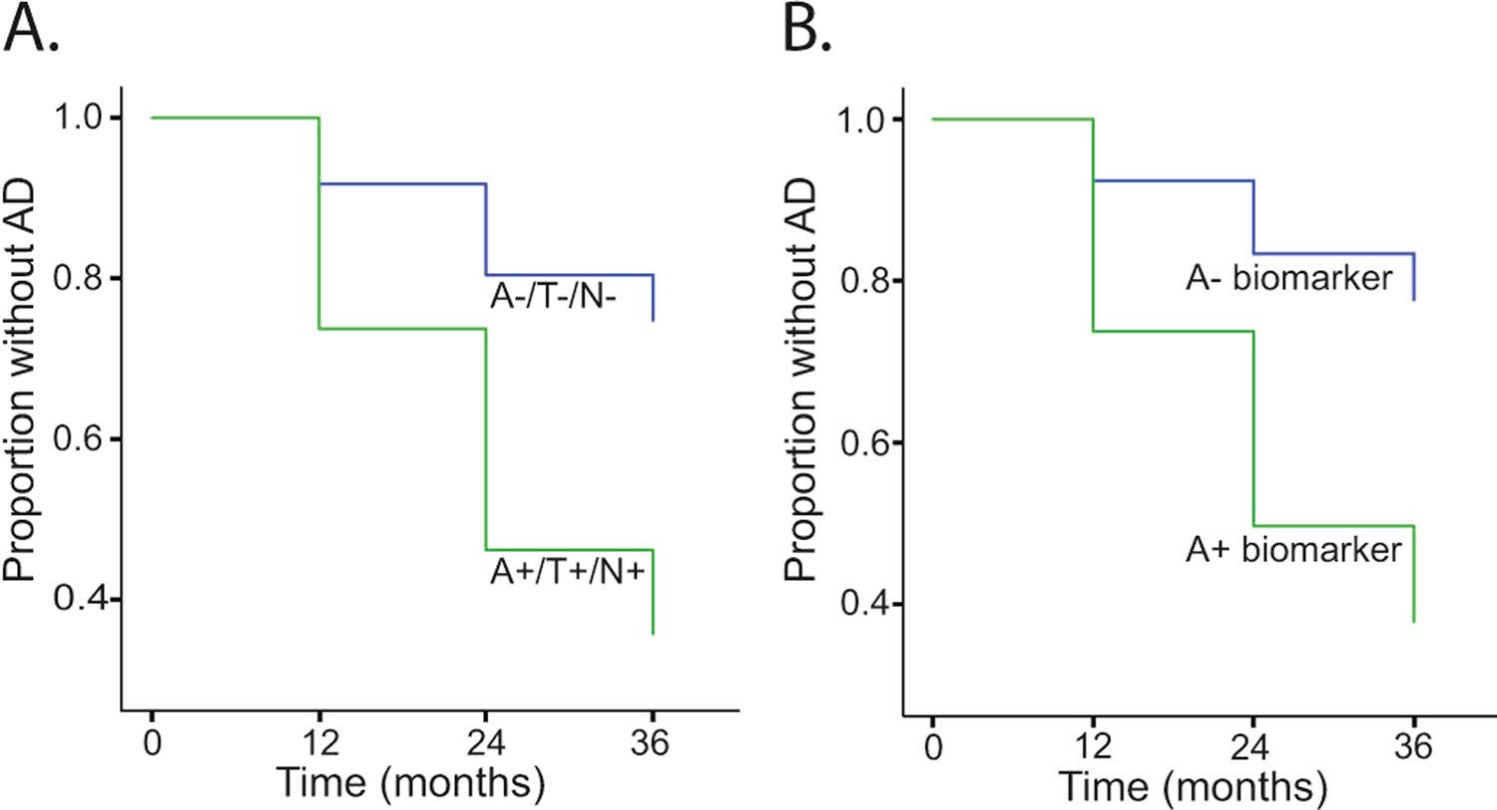
Survival curves of different A/T/N groups in MCI Showing survival curves that illustrate time of progression to AD for participants with MCI at baseline with either (A) an A−/T−/N− (blue; n = 30; mean duration = 28.8 months, SD = 9.8) or an A+/T+/N+ (green; n = 63; mean duration = 25.5 months, SD = 9.8) pattern, or (B) a negative (blue; n = 40; mean duration = 28.8 months, SD = 10.1) or a positive (green; n = 114; mean duration = 25.0, SD = 9.9) “A” biomarker (irrespective of “T” and “N” classification).

The descriptive results of the A/T/N classification are displayed in Fig. 2 (see also supplementary material for specific baseline information on the comparison between A−/T−/N− or A+/T+/N+ MCI subjects). Overall, the overlap between amyloid pathology, tangle pathology, and neurodegeneration increased with disease progression, being extensive in MCI-P and AD, and inferior in MCI-S and HC. In particular, participants with a positive “A” biomarker were more highly represented in AD (92%) and MCI-P (91%) than in MCI-S (59%) and HC (39%). Of the 114 MCI-participants with positive “A”, 67 progressed to AD (59%), which correspond to an incidence rate of 196 per 1000 individuals with MCI that progress yearly to AD (95% CI, 154–238). Furthermore, an overall positive A/T/N classification (i.e., A+/T+/N+) was more common in AD (63%) and MCI-P (54%) than in MCI-S (28%) and HC (12%). In contrast, an overall negative A/T/N classification (i.e., A−/T−/N−) was more common in HC (43%) and MCI-S (31%) than in MCI-P (8%) and AD (4%). As illustrated in Fig. 3, being classified as MCI at baseline with an A+/T+/N+ (n = 63) score increased the risk (HR = 3.54) of progressing to AD compared with an A−/T−/N− (n = 30) score (95% CI = 1.50–8.37; p < 0.01). In addition, being classified with a positive “A” (n = 114) biomarker (irrespective of T and N classification) increased the risk (HR = 3.84) of progressing to AD compared with a negative “A” (n = 40) biomarker (95% CI = 1.76–8.37; p < 0.01). As a control analysis, we added the baseline scores for AVLT delayed recall as a covariate to the model. The result showed that the HR for the “A” biomarker was still high (3.07) and significant (p < 0.01).

### Characteristics of atrophy patterns in MCI

The demographics and clinical variables for different atrophy patterns in MCI are displayed in Table 2. There was a significant group difference regarding age (F_7,146_ = 3.66, p < 0.01) but not education (F_7,146_ = 1.73, p = 0.11). Post-hoc analyses showed that MCI-S with minimal-atrophy were younger than both MCI-S with Limbic-predominant atrophy and MCI-P with typical AD. The neuropsychological testing revealed group differences for both global cognitive performances measured with MMSE (F_7,146_ = 2.71, p = 0.01) and episodic memory measured with AVLT delayed recall (F_7,146_ = 3.33, p < 0.01). The significant effects on MMSE and AVLT remained after including age and education as covariates. Post-hoc analyses showed no significant group differences on MMSE, but MCI-S with minimal-atrophy performed significantly better on AVLT than MCI-P with limbic-predominant atrophy. There were also group differences regarding the CSF biomarkers Aβ_42_ (F_7,146_ = 3.76, p < 0.01), p-tau (F_7,146_ = 3.04, p < 0.01), and t-tau (F_7,146_ = 3.21, p < 0.01). Post-hoc analyses showed that Aβ_42_ levels were significantly higher in MCI-S with hippocampal-sparing pattern than both MCI-P with minimal-atrophy and MCI-P with a typical AD pattern. For p-tau, the levels were significantly lower for MCI-S with limbic-predominant pattern than both MCI-P with minimal-atrophy, and MCI-P with the hippocampal-sparing pattern. For t-tau, the levels were significantly lower for MCI-S with limbic-predominant atrophy than MCI-P with minimal-atrophy. Of all atrophy groups, MCI-P with minimal-atrophy had the lowest levels of Aβ_42_, as well as the highest levels of p-tau and t-tau. The distribution regarding gender (X^7^ = 4.16, p = 0.76) and APOE ε4 (X^7^ = 7.47, p = 0.38) were rather similar between groups.

**Table 2:** Demographics and clinical variables for the atrophy patterns in MCI.

	MCI-SM- A (n = 31)	MCI-S L-P (n = 21)	MCI-ST-AD (n = 10)	MCI-S H-S (n = 18)	MCI-P M-A (n = 21)	MCI-P L-P (n = 25)	MCI-P T-AD (n = 19)	MCI-P H-S (n = 9)
Age**	70,81 (7,05)	76,94 (5,15)	77,16 (8,37)	76,49 (6,38)	71,17 (7,89)	73,97 (6,30)	77,26 (5,66)	77,95 (8,76)
Education	15,84 (2,48)	17,00 (2,61)	17,70 (2,26)	14,83 (3,60)	15,05 (3,37)	16,24 (3,01)	15,95 (1,99)	14,89 (4,57)
MMSE*	27,58 (1,52)	26,71 (1,45)	25,90 (1,66)	27,78 (1,70)	26,67 (2,01)	26,36 (1,58)	26,68 (2,11)	26,00 (1,23)
AVLT del**	3,87 (3,45)	2,81 (3,12)	1,90 (2,13)	3,78 (3,61)	1,48 (1,97)	1,36 (2,20)	1,47 (2,20)	1,33 (1,73)
Aβ_42_**	172,43 (56,12)	168,75 (57,49)	162,29 (50,82)	206,73 (60,01)	137,45 (22,52)	150,03 (32,12)	145,22 (48,80)	168,92 (51,51)
Abnormal Aβ_42_	61%	67%	70%	39%	100%	92%	84%	78%
p-tau**	33,19 (17,90)	27,62 (15,30)	32,30 (15,38)	28,56 (12,36)	45,05 (14,82)	34,72 (12,93)	42,79 (22,02)	39,89 (15,23)
Abnormal p-tau	65%	52%	70%	50%	95%	84%	79%	78%
t-tau**	88,16 (43,58)	79,65 (47,87)	99,80 (43,67)	81,82 (37,79)	129,77 (54,98)	93,93 (30,19)	124,36 (76,22)	125,64 (61,91)
Abnormal t-tau	39%	24%	36%	40%	67%	44%	58%	67%
APOE ε4Positive N/Y	17/14	12/9	6/4	11/7	8/13	8/17	7/12	4/5
Gender W/M	13/18	4/17	3/7	6/12	9/12	9/16	6/13	4/5

Means, parentheses = standard deviations. MCI-S = MCI participants that are clinically stable across time. MCI-P = MCI participants that progress to AD at follow-up. MMSE = Mini Mental State Examination. AVLT = Auditory Verbal Learning Test. N = No/Y = Yes. W = Women/M = Men. M-A = Minimal atrophy. L-P = Limbic Predominant. T-AD = Typical Alzheimer’s disease. H-S = Hippocampal-sparing. p-tau = phosphorylated tau. t-tau = total tau. *p < 0.05. **p < 0.01.

### A/T/N classification on atrophy patterns in MCI

**Figure 4:**
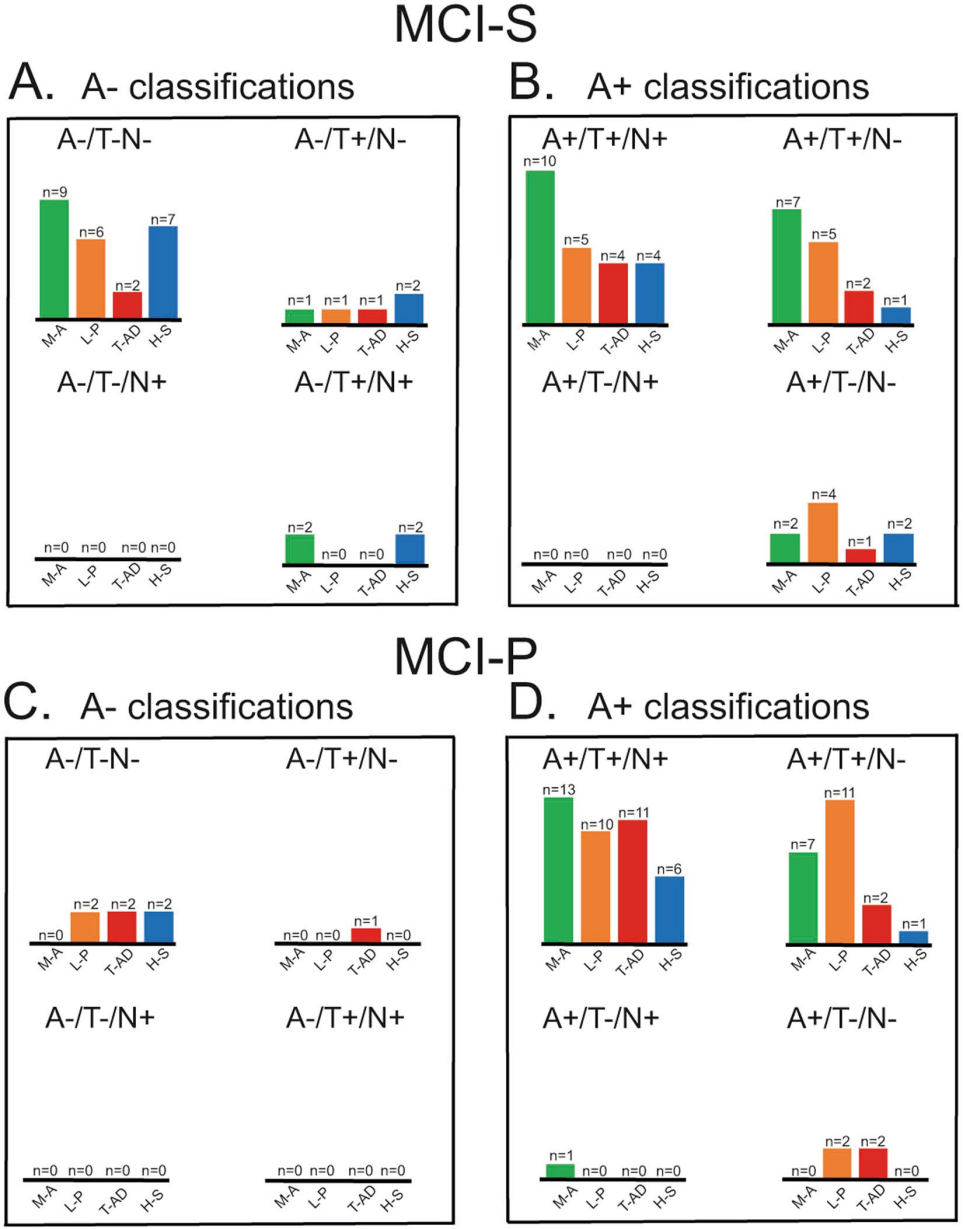
Number of A/T/N classified MCI-participants with different atrophy patterns Number of participants with MCI-S and an A- classifications (**A**), MCI-S and an A+ classification (**B**), MCI-P and an A- classifications (**C**), and MCI-P and an A+ classification (**D**) pattern. MCI-S = MCI participants that are clinically stable across time. MCI-P = MCI participants that progress to AD. A = Aβ biomarker. T = tau pathology biomarker. N = the biomarker of neurodegeneration. M-A = Minimal atrophy. L-P = Limbic Predominant. T-AD = Typical Alzheimer’s disease. H-S = Hippocampal-sparing.

The A/T/N distribution of different atrophy patterns in MCI is illustrated in Fig. 4. Overall, amyloid positive (A+) individuals showed a staging pattern where the A+/T+/N+ pattern was most common, followed by the A+/T+/N− pattern, and finally the A+/T−/N− pattern was less common. Only one individual had an A+/T−/N+ pattern. In MCI-S, 28% (n = 23) of those that had the minimal-atrophy or hippocampal-sparing pattern also had a negative “A” biomarker. The equivalent number for MCI-P was 3% (n = 2), and the proportion comparison between MCI-S and MCI-P was significant (X^1^ = 25.00, p < 0.01). In addition, no individual in the MCI-P group with minimal-atrophy had a negative “A” biomarker, whereas the equivalent number in MCI-S was 15% (n = 12). An overall positive A/T/N classification (i.e., A+/T+/N+) was common in all MCI-P atrophy patterns. Notably, the A+/T+/N+ pattern in MCI-P was more frequent in the minimal-atrophy pattern (n = 14) than the Limbic-predominant (n = 10) and the Typical AD pattern (n = 11) subgroups.

**Figure 5:**
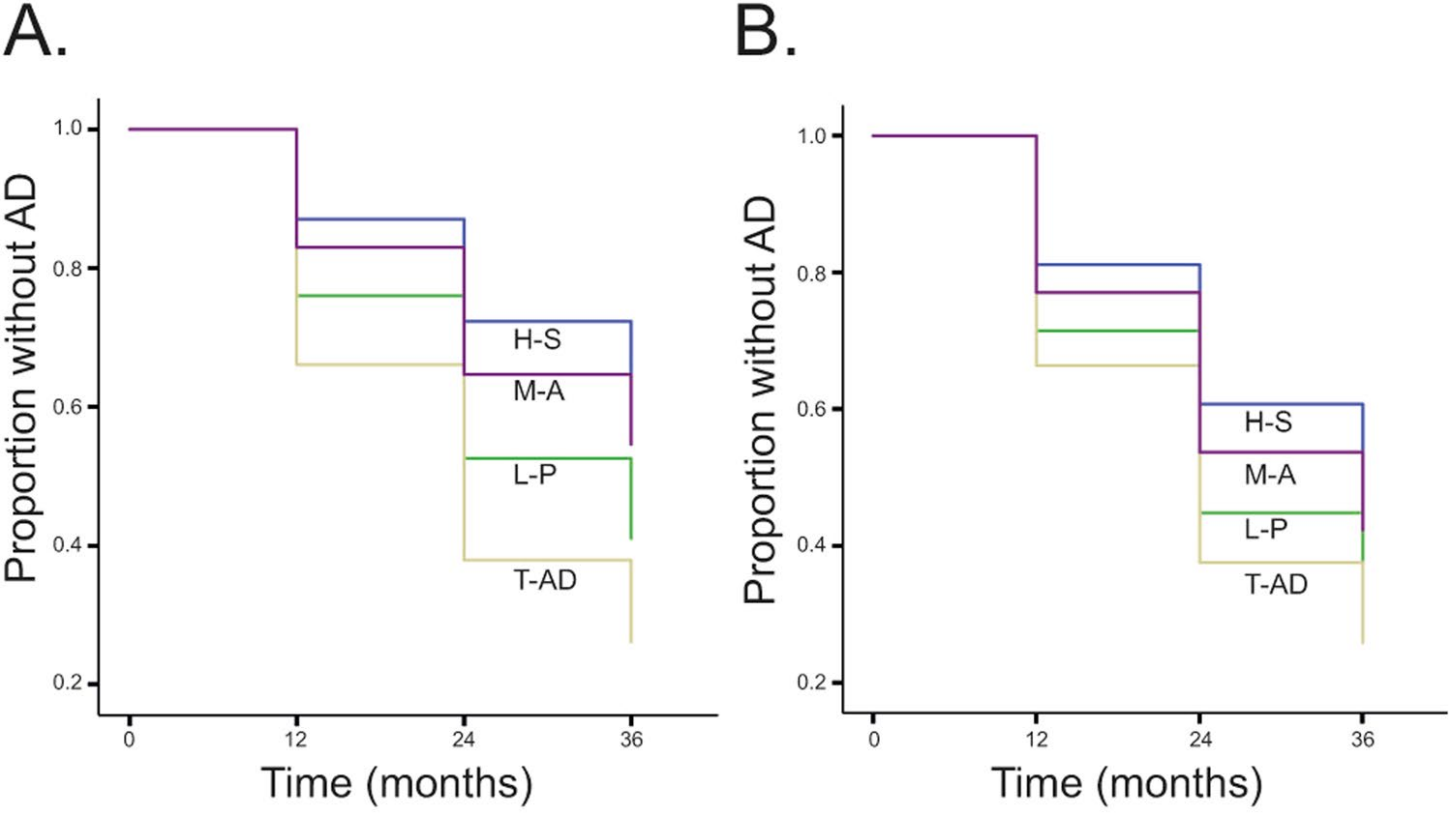
Survival curves of different brain atrophy patterns in MCI (**A**) Showing survival curves that illustrate time to AD for participants with MCI at baseline with either M-A = Minimal atrophy (purple), L-P = Limbic Predominant atrophy (green), T-AD = Typical Alzheimer’s disease atrophy (beige), or H-S = Hippocampal-sparing pattern (blue). (**B**) Showing survival curves as in A, but only including individuals with A+ biomarkers in the model.

As illustrated in Fig. 5a, a survival curve comparison between the different atrophy patterns at baseline, showed that the group with typical AD pattern (n = 29) had a HR of 2.99 times (p < 0.01) that of the group with hippocampal-sparing pattern (n = 27; i.e., reference group due to slowest disease progression). In addition, the group with limbic-predominant pattern (n = 46) had a HR of 1.98 times (p = 0.079) that of the group with hippocampal-sparing pattern. When adding the “A” biomarker information (i.e., if the participant had an A− or an A+ classification) and age (that was significant in the ANOVA) to the model, the “A” biomarker covariate had a HR of 3.40 (p < 0.01) indicating that exposure to the covariate increased the risk of progressing to AD. The age covariate had a HR of 1.00 (p = 0.87) indicating no effect on the outcome. In addition, when only including individuals with A+ biomarkers in the model, a survival curve comparison (Fig. 5b) between different atrophy patterns at baseline, showed that the group with typical AD pattern had a HR of 1.96 times that of the group with hippocampal-sparing pattern, but it did not render in statistical significance (p = 0.14).

## Discussion

We recognize that the A/T/N biomarker system is a useful classification scheme in the MCI population. Importantly, the addition of different brain atrophy patterns accumulated the diagnostic substrate and increased knowledge of the clinical trajectories. This combined approach could be important for increasing certainty in the diagnostic and prognostic procedures in MCI, and therefore also supportive in targeting good candidates for interventions. We will first discuss the A/T/N classification scheme assessed in individuals with MCI and then discuss the impact of brain atrophy patterns when added to the A/T/N classification, with a special focus on variability in the “N” domain.

### The A/T/N classification

We showed a progression rate from amnestic MCI to AD (47%) which is consistent (48%) with a previous study with the same follow-up time of 36-months^25^. In addition, we showed that 58% of the MCI-participants with a positive “A” biomarker progressed to AD which is comparable with a previous study showing that 57% of an MCI-population with abnormal Aβ_42_ progressed to AD during a mean follow-up of 2.2 years^26^. Notably, being classified as MCI with a positive “A” biomarker increased the risk of progressing to AD during a 36-month period in comparison with being classified with a negative “A” biomarker. The effect was evident even after controling for cognitive baseline status. This resembles models of AD-related neurodegeneration, in which amyloid deposition acts as the trigger of further disease progression^27^. In addition, the A/T/N classification showed a stepwise increase in numbers of A+/T+/N+ profiles from HC (12%), via MCI-S (29%), to MCI-P (54%), and finally the highest number for AD (63%). Thus, the more advanced stage of the disease, the higher was the prevalence of positive A/T/N biomarkers, as it was a comorbidity of pathophysiological processes. In addition, the MCI subjects with an overall positive A/T/N pattern had a worse episodic memory performance compared with those with an overall negative A/T/N pattern which might reflect a later stage of MCI for A+/T+/N+. Interestingly, we showed that MCI-P had similar A/T/N classification patterns to the group with AD. A positive “A” biomarker was represented in more than ninety percent in both MCI-P and AD. However, a positive “A” biomarker was also present in MCI-S (59%) and HC (39%), but to a lesser degree. Thus, there was a considerable amount of individuals in HC and MCI-S with evidence of AD-like pathology. However, Aβ_42_ deposition increases with age and is also apparent in healthy individuals^28,29^. Recently, an A/T/N classification in cognitively normal individuals showed that the A+/T+/N+ prevalence increased continuously with age^30^. Notably, considering all groups, only one individual had the A+/T−/N+ profile. This might reflect an individual in an early stage of preclinical AD (A+/T−) in conjunction with non-AD pathology (N+)^6^. Thus, in our A/T/N classification of MCI, the CSF biomarker patterns seems to generally follow the order of A+, then T+, and finally N+. As in healthy aging individuals^30^, the biomarker sequence of A+/T−/N− to A+/T+/N− to A+/T+/N+ is also noted in the current study of MCI and might represents the pre-dementia AD staging pattern^7^. However, ADNI is a highly selective cohort and the A+/T−/N+ profile might be more commonly occurring in clinical settings with more diverse pathology and clinical trajectories. Importantly, longer follow-ups are needed to increase knowledge on the staging evolvement in healthy individuals as well as in MCI to confirm the A/T/N progression.

Furthermore, we showed that MCI with a negative “A” biomarker was more common in MCI-S than in MCI-P. Despite that, we reported that seven individuals with MCI and a negative “A” biomarker progressed to AD within 36 months. Of those seven, six had an A−/T−/N− pattern, and one an A−/T+/N− pattern. Interestingly, when we added information on atrophy patterns to those profiles, we demonstrated that five individuals had an abnormal MTA score (two individuals with a limbic-predominant pattern and three with a typical AD pattern), and the additional two had abnormal cortical atrophy (both with a hippocampal-sparing pattern). Thus, the biomarker profiles of these individuals might be related to suspected non-AD pathophysiology (SNAP)^6,27^, that has previously been suggested to target individuals without evidence of amyloid accumulation but with evidence of neuronal injury. Possibly, such a pattern might also resemble neurodegeneration due to non-AD pathology^30^. In addition, no MCI individual with an A−/T−/N− profile and minimal-atrophy progressed to AD within the studied time-frame. Thus, an MCI diagnosis with an overall negative A/T/N classification in addition to a pattern of minimal-atrophy does not seem to represent MCI due to AD^31^ or prodromal AD, and the clinical cognitive symptoms are probable due to other underlying causes^8^. All these findings suggest that in MCI individuals with a negative “A” biomarker, it is crucial to investigate the patterns of atrophy on MRI as well, which may have higher diagnostic utility than the CSF T-Tau levels.

### The addition of brain atrophy patterns

Notably, the A+/T+/N+ pattern was more common in the MCI-P groups with minimal atrophy and hippocampal sparing pattern than the MCI-P groups with limbic predominant and typical AD patterns. The minimal atrophy and hippocampal sparing groups had lower levels of education, and the minimal atrophy group was also younger than the other groups, as recently reported in AD dementia^32^. In addition, MCI-P with minimal atrophy had the lowest levels of Aβ_42_ and the highest levels of p-tau and t-tau. This is in conjunction with what has previously been reported in groups with minimal atrophy in an AD population^13^. Also, the step-wise addition of p-tau (as a neurodegeneration marker) to cognitive measures has previously shown to improve risk stratification compared to hippocampal volumes (derived from MRI) in subjects with MCI^33^. Individuals with MCI and a minimal atrophy pattern in conjunction with abnormal CSF (A+/T+/N+) could therefore be good candidates for interventions due to their absence of irreversible brain atrophy, together with young age, less cognitive impairment at baseline (i.e., AVLT delayed), and slow cognitive decline across time^13^.

Additionally, as suggested by Jack *et al*.^6^, we described the “N” category with only one biomarker (t-tau) in the current study. Another established marker to describe the “N” category is brain atrophy derived from MRI. When comparing the “N” classification with the atrophy patterns, several individuals with MCI showed inconsistency regarding the “N” binary classification and the MRI character of atrophy. For example there were individuals with MCI-P and A+/T+/N+ in conjunction with minimal atrophy (i.e., N− according to the visual rating scales). This highlights an incongruity between CSF t-tau and brain atrophy in MRI. Those conflicting results have been emphasised previously, showing that neurodegeneration biomarkers are modestly correlated^27^. In the absence of overt neuronal injury this might be related to increased neuronal secretion of tau in response to amyloid pathology, and that CSF t-tau and p-tau concentrations do not reflect neurodegeneration and tangle pathology directly^34^. These neurons may eventually accumulate tangle pathology and degenerate, but that would become evident in MRI many years later. In addition, this emphasises that individuals with MCI display heterogeneous neurodegeneration patterns as reflected on both MRI^35^, and CSF^36^, and that different biomarkers might become abnormal at different stages of the disease progression, or never coincide^37^.

The MCI individuals with a positive “A” biomarker in conjunction with amnestic impairment should be considered as prodromal AD according to the IWG criteria^8^, and as MCI due to AD according to the NIA-AA criteria^31^. In agreement, the addition of atrophy patterns to the “A” biomarker information, at least to some degree, enhanced confidence to the MCI prognoses in the current study. The progression rate from MCI to AD varies and annual rates between 4–36% have been reported^38^. Thus, we cannot with certainty confirm that MCI-S individuals in the current study will progress to AD. However, the MCI-S individuals with a positive “A” biomarker and AD-like atrophy patterns are likely to progress in respect to previous reports. For example, higher rates of AD-like atrophy has been reported in progressive MCI^39,40^, and amyloid positive MCI individuals have been shown to be more likely to progress to AD than amyloid negative MCI individuals^41^. However, further longitudinal large-scale clinical studies that examine atrophy patterns in conjunction with A/T/N classifications are needed to increase knowledge on different MCI pathways. The current study shows for the first time that this joint strategy holds high promise.

### Limitations and future prospects

Since MCI individuals in ADNI are amnestic at entry, a future prospect is to adapt the A/T/N classification scheme and atrophy patterns to MCI participants with non-amnestic clinical presentations. As shown previously, the nature of memory impairment might differ due to the influence of non-memory cognitive functions on memory performance across atrophy patterns in AD^13^. In the current study, MCI and AD are only represented by amnestic deficits which narrows the spectra of atypical AD-related topographies, also due to the unresolved question whether AD must be represented solely by amnestic impairment^42^. In addition, the A/T/N classification scheme needs to be further validated, especially in community samples with MCI subjects. This would make it possible to draw firmer conclusions on the clinical significance of the scheme and first thereafter be able to be applied on MCI subjects in general. Thus, research studies in large-scale clinical settings are important to increase knowledge on this and other related topics.

This study has some additional limitations. Several individuals have CSF values close to the cut-offs. In clinical settings, the values close to the cut-offs have to be carefully interpreted and handled in relation to additional clinical information, as well as additional biomarker information. We only followed the participants for 36 months after their initial diagnosis, and a longer follow-up might have increased the number of MCI participants progressing to AD^43^. In addition, the sub-group categorization focusing on atrophy patterns in MCI partly rendered in small sample sizes, and a future prospect would therefore be to maximise sample sizes to increase the possibilities for stronger generalizations to the MCI-population.

## Conclusions

We conclude that the A/T/N classification scheme is easy and adaptable on individuals with MCI, and that visual rating scales can be used to identify AD-related atrophy patterns also in MCI. We presented that no individual with MCI progressed to AD if they had a negative “A” biomarker in conjunction with minimal atrophy. In contrast, several individuals with MCI progressed to AD if they had a positive “A” biomarker in conjunction with minimal atrophy. In addition, many individuals with MCI showed inconsistency in the neurodegeneration domain (“N”), reflecting an incongruity between CSF t-tau levels and brain atrophy on MRI. Thus, we propose that the A/T/N biomarker system should be applied and interpreted together with information about brain atrophy patterns. This joint strategy shows promising sings for increasing certainty in the diagnostic and prognostic procedures in MCI, and can also be supportive in targeting suitable candidates for interventions. Accordingly, implementation in the clinical routine could be justified.

## Electronic supplementary material

Supplementary information
