# Supplementary material for: The A/T/N biomarker scheme and patterns of brain atrophy assessed in mild cognitive impairment
Source: Sci Rep. 2018 May 30;8:8431. doi: 10.1038/s41598-018-26151-8 (PMC5976713; doi:10.1038/s41598-018-26151-8)
Supplement: Supplementary file 1 — Supplementary information [file 41598_2018_26151_MOESM1_ESM.pdf]

The A/T/N biomarker scheme and patterns of brain atrophy assessed in mild cognitive impairment

Authors: Urban Ekman*, PhD[1] Daniel Ferreira, PhD[1] Eric Westman, PhD[1,2]

[1] Division of Clinical Geriatrics, Department of Neurobiology, Care Sciences, and Society, Karolinska Institutet, Stockholm, Sweden.

[2] Department of Neuroimaging, Centre for Neuroimaging Sciences, Institute of Psychiatry, Psychology and Neuroscience: King's College London, London, UK.

ADNI centres:

Johns Hopkins University

Washington University, St. Louis

University of California, Los Angeles

University of Pennsylvania

Cleveland Clinic Lou Ruvo Center for Brain Health

Sunnybrook Health Sciences Centre

Parkwood Hospital

University of California, San Diego

University of Kansas

Dent Neurologic Institute

McGill University / Jewish General Hospital Memory Clinic

Rush University Medical Center

Baylor College of Medicine

Duke University Medical Center

Wein Center for Clinical Research

Indiana University

St. Joseph's Health Center – Cognitive Neurology

Banner Alzheimer's Institute

New York University Medical Center

Mayo Clinic, Jacksonville

Mount Sinai School of Medicine

University of Michigan, Ann Arbor

University of British Columbia, Clinic for AD & Related

University of Wisconsin

Oregon Health and Science University

Northwestern University

Boston University

Case Western Reserve University

Emory University

University of Pittsburgh

Brigham and Women's Hospital

University of Alabama, Birmingham

Medical University of South Carolina

University of California, Irvine

Howard University

University of California, Davis

Rhode Island Hospital

Mayo Clinic, Rochester

Nathan Kline Inst. for Psychiatric Rsch

University of Rochester Medical Center

University of California, Irvine (BIC)

The Weill Cornell Memory Disorders Program

Georgetown University

University of California, San Francisco

Banner Sun Health Research Institute

Premiere Research Institute

Butler Hospital Memory and Aging Program

Dartmouth Medical Center

Ohio State University

University of Southern California

University of Iowa

Wake Forest University Health Sciences

University of Kentucky

University of South Florida, Tampa

Columbia University

Yale University School of Medicine

University of Texas, Southwestern MC

Stanford / PAIRE

Albany Medical College

Table:

Baseline characteristics for MCI subjects with either A-/T-/N- or A+/T+/N+

	MCI A-/T-/N- (n = 30)	MCI A+/T+/N+ (n = 63)
**Number of MCI-S/MCI-P****	24/6	23/40
**Age**	74,97 (7,32)	73,28 (7,33)
**Education (years)**	15,87 (3,16)	15,87 (2,97)
**MMSE**	27,37 (1,61)	26,78 (1,84)
**AVLT Del****	4,00 (3,43)	1,71 (2,61)
**APOE ε4 positive Y/N****	4/26	44/19
**Gender W/M**	7/23	25/38

Means, parentheses = standard deviations. A- = CSF Aβ normal. A+ = CSF Aβ abnormal. T- = CSF p-tau normal. T+ = CSF p-tau abnormal. N- = CSF t-ta u normal. N+ = t-tau abnormal. MCI-S = MCI participants that are clinically stable across 36 months of follow-up. MCI-P = MCI participants that progress to AD within 36 months of follow-up. MMSE = Mini Mental State Examination. AVLT = Auditory Verbal Learning Test. N = No/Y = Yes. W = Women/M = Men. * = $p < 0.05$. ** = $p < 0.01$.
